# Practical Departments

**Published:** 1905-09-23

**Authors:** 


### PRACTICAL DEPARTMENTS.

### A NURSE'S LAMP.

A VERY convenient and useful lamp bears the name of The Safety Parabolic Reflector Lamp, and is supplied by Messrs. Bruce, 232 Borough High Street, S.E. It is intended to burn ordinary lamp-oil, and gives a brilliant light. It is fitted with a reflector, which can be moved in any direction, and has a safety tube. The oil is introduced by means of a screw filler at the side. A handle is attached, so that it forms a safe and serviceable portable light. It is exactly what the night nurse wants, and will be found generally useful in the sick-room. The lamps are neatly and simply made in English brass, price 4s. 6d., or, in nickel plate, price 5s. 6d.

